# Exploring the Potential of Energy-Based Therapeutics (Photobiomodulation/Low-Level Laser Light Therapy) in Cardiovascular Disorders: A Review and Perspective

**DOI:** 10.7759/cureus.37880

**Published:** 2023-04-20

**Authors:** Vijay Durga Pradeep Ganipineni, Sai Dheeraj Gutlapalli, Idavalapati Ajay Sai Krishna Kumar, Potru Monica, Moparthi Vagdevi, Tamalapakula Samuel Sowrab

**Affiliations:** 1 Department of General Medicine, SRM Medical College Hospital and Research Center, Chennai, IND; 2 Department of General Medicine, Andhra Medical College/King George Hospital, Visakhapatnam, IND; 3 Department of Internal Medicine, Richmond University Medical Center - Mount Sinai Health System/Icahn School of Medicine at Mount Sinai, Staten Island, USA; 4 Internal Medicine Clinical Research, California Institute of Behavioral Neurosciences & Psychology, Fairfield, USA; 5 Department of Medicine, Guntur Medical College, Guntur, IND; 6 Department of Medicine, Dr. Pinnamaneni Siddhartha Institute of Medical Sciences and Research Foundation, Vijayawada, IND; 7 Department of Medicine, Katuri Medical College and Hospital, Guntur, IND

**Keywords:** :heart failure, light-emitting diode (led), devices in cardiology, cardiology devices, cardiology research, wound healing and tissue repair, cardiac remodelling, cardiovascular disease, photobiomodulation, low-level laser therapy

## Abstract

Based on the review of the literature, this article examines the potential therapeutic benefits of photobiomodulation therapy (PBMT) or low-level laser therapy (LLLT) for the treatment of cardiovascular disorders. The methodology involved searching PubMed, Google Scholar, and Central databases for relevant articles published from inception till date. The articles included in this review were preclinical and clinical studies investigating the effects of PBMT and LLLT on the heart. The article summarizes the findings of nineteen studies investigating the effects of PBMT and LLLT on various parameters related to heart failure (HF) and myocardial infarction (MI), including inflammation, oxidative stress, angiogenesis, cardiac function, and remodeling. The studies suggest that PBMT and LLLT have potential therapeutic benefits for the treatment of cardiovascular diseases and could be used in combination with traditional pharmacological therapies to enhance their effects or as a stand-alone treatment for patients who are not responsive to or cannot tolerate traditional therapies. In conclusion, this review article highlights the promising potential of PBMT for the treatment of HF and MI and the need for further research to fully understand its mechanisms of action and optimize treatment protocols.

## Introduction and background

Introduction

Photobiomodulation (PBM) or low-level laser therapy (LLLT) is a non-invasive therapeutic approach that uses low-level light sources, typically in the form of low-power lasers or light-emitting diodes (LEDs), to stimulate cellular responses and promote healing. The process involves the absorption of red or near-infrared (NIR) light by specific cellular components, mainly the mitochondria, resulting in a cascade of biological reactions that enhance cellular function and overall tissue health. Photobiomodulation (PBM) or low-level laser therapy (LLLT) was demonstrated in numerous in vitro studies to exhibit unique biological effects with a dose-dependent cellular action mechanism [1]. Since its inception in the year 1967, more than 400 randomized, double-blinded clinical trials, some featuring placebo controls, have been published for various employments [2]. The intricate biological mechanisms responsible for LLLT/PBM's therapeutic effects have not been entirely understood, and these mechanisms might differ among cell types and tissue conditions, such as healthy versus stressed or hypoxic states. Nevertheless, both laboratory and clinical investigations indicate that LLLT/PBM effectively diminishes inflammation, prevents fibrosis [3-9], alleviates pain, and enhances overall organism function when applied appropriately [1,10-12].

Emerging evidence suggests that PBM primarily acts on Cytochrome c Oxidase (CcO) in the mitochondrial respiratory chain, facilitating electron transport and subsequently increasing adenosine triphosphate (ATP) production by boosting the transmembrane proton gradient [13]. As ATP is the universal energy source for all biological activities in living cells, even a minor upsurge in ATP levels can improve bioavailability for cellular metabolism functions [1]. Furthermore, red or NIR light absorption may cause a brief, transient surge of reactive oxygen species (ROS), followed by an adaptive decrease in oxidative stress [1]. The low ROS concentrations activate numerous cellular processes, including transcription factors such as nuclear factor kappa B (NF-κB), which in turn upregulate stimulatory and protective genes [14]. These genes produce fibroblast growth factors, cytokines and chemokines involved in tissue regeneration.

**Figure 1:**
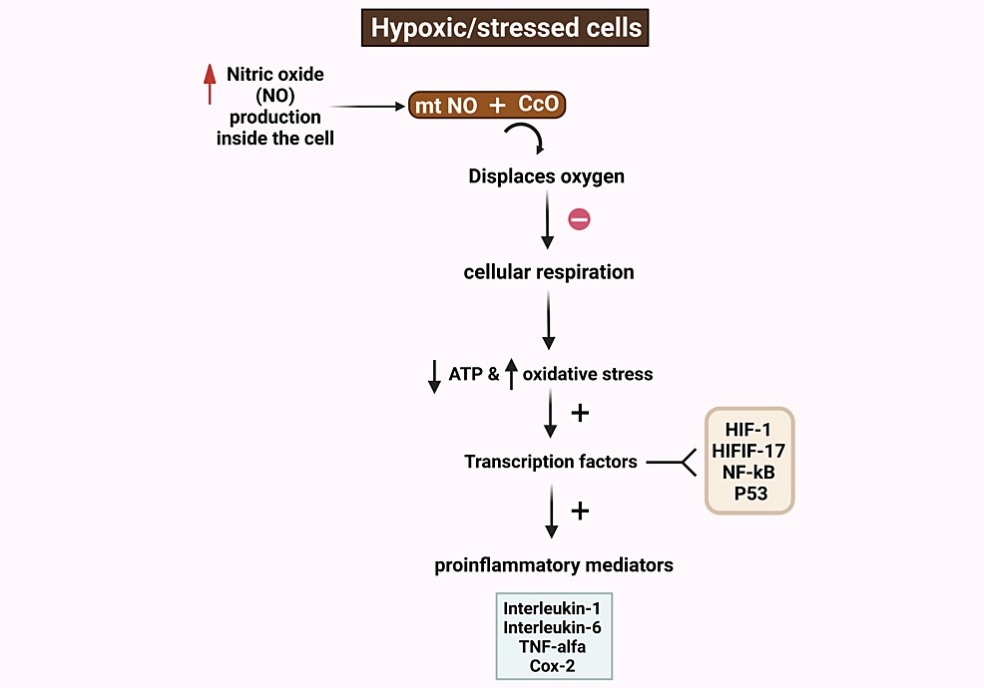
The intracellular events in a stressed/hypoxic cell The figure depicts the intracellular events that occur in a stressed or hypoxic cell. The figure shows that in a hypoxic cell, mitochondria generate excess nitric oxide (NO), that binds to Cytochrome c Oxidase (CcO) and displaces oxygen. The displacement of oxygen from CcO leads to inhibited cellular respiration, reduced ATP generation and increased oxidative stress. This state activates various intracellular signalling pathways and transcription factors such as redox factor-1, hypoxia-inducible factor-1, HIF-like factor 17, activator protein-1, nuclear factor-kB, p53, activating transcription factor/cyclic adenosine monophosphate (cAMP)-response element-binding protein (ATF/CREB), inducing the downstream production of inflammatory mediators like interleukins IL-1 and IL-6, tumor necrosis factor-alpha, cyclooxygenase (COX)-2, and prostaglandin E2. The inflammatory milieu delays or halts cellular repair and tissue healing. ATP (Adenosine Triphosphate), HIF (Hypoxia Inducible Factor), HIFLF (Hypoxia Inducible Factor Like Factor), NF (Nuclear Factor), TNF (Tumor Necrosis Factor), Cox (Cyclooxygenase)

In hypoxic cells or stressed cells, mitochondria generate nitric oxide (NO), that binds to CcO and displaces oxygen [15]. The result of this binding leads to inhibited cellular respiration, reduced ATP generation and increased oxidative stress [16]. This state activates various intracellular signaling pathways and transcription factors such as redox factor-1, hypoxia-inducible factor (HIF)-1, and HIF-like factor 17, activator protein-1, nuclear factor-kB, p53, activating transcription factor/cyclic adenosine monophosphate (cAMP)-response element-binding protein (ATF/CREB), inducing the downstream production of both inflammatory mediators like interleukins IL-1 and IL-6, tumor necrosis factor-alpha, cyclooxygenase (COX)-2, and prostaglandin E2 and anti-inflammatory mediators like Transforming Growth Factor-beta and IL-10 (Figure 1) [16,17,18,19].

**Figure 2:**
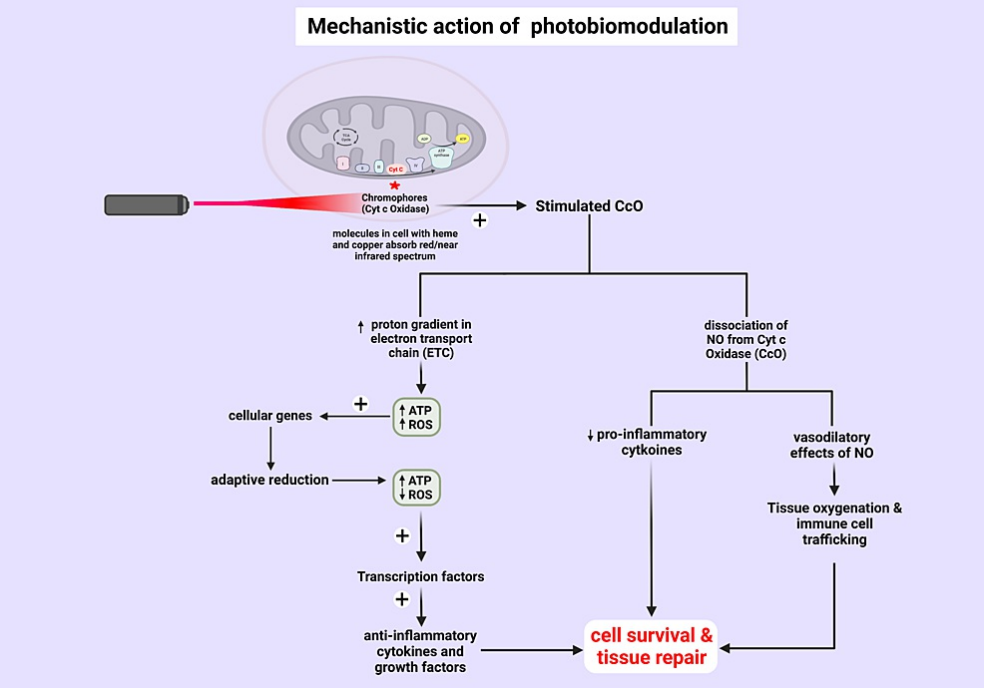
Mechanism of action of photobiomodulation in biological tissue The mechanism of action of PBMT/LLLT for promoting cell repair involves the mitochondrial electron transport chain (ETC). The chromophores (e.g. Cytochrome c Oxidase) are iron/copper-containing molecules that can absorb red/infrared light spectrum. Upon absorbing the incoming energy from a light-emitting device, Cytochrome c Oxidase (CcO) a critical enzyme in ETC gets activated. This results in the dissociation of NO from CcO which in turn reverses the hypoxic cell cascade explained in Figure 1. Free NO causes vasodilation increasing tissue oxygenation. On the other hand, CcO activation also increases ATP production and reduces ROS. The net effects promote cell survival and tissue repair. NO (Nitric Oxide), ROS (Reactive Oxygen Species), PBMT (Photobiomodulation Therapy), LLLT (low-level laser therapy), ATP (Adenosine Triphosphate)

Evidence indicates that administering LLLT/PBM with appropriate parameters to stressed cells can dissociate NO from its competitive binding to CcO, increase ATP production, and restore the balance between pro and antioxidant mediators, reducing oxidative stress [20]. For instance, LLLT/PBM has been demonstrated to attenuate ROS production in neutrophils [21] and reduce ROS in an animal model of traumatic tissue injury [22]. Additionally, PBM has been found to decrease the generation of tumor necrosis factor alpha (TNF-α) and increase IL-10, an anti-inflammatory cytokine, in a model of acute lung inflammation [23]. Furthermore, NO's vasodilatory properties [24] can enhance blood supply to illuminated tissue, while LLLT-mediated vascular regulation increases tissue oxygenation and immune cell trafficking [1]. These two effects may contribute to promoting wound repair and regeneration (Figure 2) [16]. The analgesic effects of PBM are likely induced by additional mechanisms beyond the increased ATP/reduced oxidative stress model. PBM with a relatively high-power density can inhibit A and C neuronal pain fibers when absorbed by nociceptors, slowing neural conduction velocity, reducing compound action potential amplitude, and suppressing neurogenic inflammation [12]. PBM has the potential to modulate almost all pathogenic mechanisms in the body (e.g., inflammation, edema, pain, fibrosis, ulceration, and neuropathy and myopathy) [1].

Consequently, LLLT has been identified as a promising approach for mitigating various cardiac pathologies [25]. Intriguing evidence suggests that LLLT's beneficial effects could persist long-term even after treatment cessation, warranting further systematic evaluation [25]. LLLT has recently been employed as an anti-inflammatory treatment in numerous diseases, including myocardial infarction (MI), where it may exert a cardioprotective effect [26]. LLLT's cardioprotective role is mediated by anti-inflammatory, antioxidant and pro-angiogenic actions [27].

Low-level laser therapy (LLLT) can alter the expression of cardiac cytokines and assists in the reversal of ventricular remodeling following myocardial injury [26]. Moreover, photobiomodulation (PBM) therapy has been shown to be effective in several age-associated chronic cardiovascular conditions, such as hypertension and atherosclerosis [28]. In this review, the focus is on examining the evidence-based investigations concerning the application of photobiomodulation therapy in cardiovascular diseases and to analyze their major propositions and recommendations.

Methodology

For this review, a comprehensive search was conducted in the PubMed, Google Scholar and Central databases using the following keywords: "low-level laser therapy," "photobiomodulation therapy," "PBM," "cardiovascular disease," "heart failure," "myocardial infarction," "cardiac remodeling," "angiogenesis," "inflammation," "oxidative stress," "aging," and "energy-based therapeutics" (Appendices). Articles published in the English language from inception till date were included. A total of nineteen relevant articles were selected for this review based on their relevance to the research question and inclusion criteria. The selected articles included randomized controlled trials, observational studies, and animal experiments. Based on the analysis of the available evidence, a perspective was presented on the potential role of energy-based therapeutics, specifically LLLT/PBM, in the prevention and management of cardiovascular disorders.

## Review

The experimental and clinical investigations were carefully considered and the mechanistic basis of low-level laser therapy, its positive influence on cardiac remodeling, reducing infarct area, restenosis prevention and other presented cardioprotective effects were examined. The summary of the salient findings of the studies are presented in Table 1.

**Table 1:** Summary of studies examining the efficacy of PBS in cardiovascular disorders LLLT (Low-Level Laser Therapy), LLLI (Low-Level Laser Irradiation), LED (Light Emitting Diode), LEDT (Light Emitting Diode Therapy), PBMT (Photobiomodulation Therapy), R/NIR (Red/Near Infrared), NO (Nitric Oxide), SOD (Superoxide Dismutase), DCFH (dichlorofluorescein), CM (Cardiomyocyte), HF (Heart Failure), ET (Exercise Test), AMI (Acute Myocardial Infarction), PCI (Percutaneous Coronary Intervention), NOS (Nitric Oxide Synthase), VEGF (Vascular Endothelial Growth Factor), IL (Interleukin), iNOS (Inducible Nitric Oxide Synthase), LSG (Left Stellate Ganglion), MI (Myocardial Infarction), miRNA (Micro RNA), ELISA (Enzyme-Linked Immunosorbent Assay), LPO (Lipid Peroxidation), NGF (Nerve Growth Factor)

Study ID	Type of study design	Focus of study	Methodology	
Biasibetti et al. 2014 [29]	Animal study	Influence of LLLT on oxidative stress and DNA damage in heart failure rats	Wistar rats were allocated into six groups and underwent a 10-day LLLT protocol on the right gastrocnemius muscle	
Blatt et al. 2016 [30]	Animal study	Effect of LLLT on stem cells, scarring, and heart function post-myocardial infarction (MI)	MI induced in pigs, followed by LLLT application to tibia and iliac bones; pigs were euthanized 90 days post-MI for analysis of scarring and heart function	
Bublitz et al. 2016 [31]	Randomized controlled trial	Acute effects of LLLT on functional capacity, perceived exertion, and blood lactate in HF patients	Patients with systolic HF were randomized into placebo LLLT (n=10) and active LLLT (n=10) groups; 6MWT performed and blood lactate determined at various time points; a multi-diode LLLT cluster probe was used	
Capalonga et al. 2016 [32]	Animal study	Effects of LEDT on functional capacity, aerobic power, and hemodynamic function in heart failure (HF) rats	Male Wistar rats allocated into Sham (n=6), Control-HF (n=4), and LEDT-HF (n=6) groups; subjected to exercise performance test (ET) twice (6 and 14 weeks after myocardial infarction); underwent phototherapy protocol for 8 weeks, 5 times/week	
Derkacz et al. 2014 [33]	Randomized controlled study	Effect of intravascular LLLT on growth factor levels in subjects undergoing percutaneous coronary intervention (PCI)	808 nm LLLT (100 mW/cm2, continuous wave laser, 9 J/cm2, illuminated area 1.6-2.5 cm2) delivered intracoronarily during PCI; 52 patients in laser group, 49 in control group; serum growth factor levels measured at various timepoints	
Derkacz et al. 2017 [34]	Randomized controlled study	Effect of LLLT on NO and endothelin-1 in patients undergoing PCI	808 nm intravascular LLLT (9 J/cm2) during PCI; 52 subjects in laser group, 49 in control group; nitrite/nitrate and endothelin-1 assessed	
Feliciano et al. 2022 [35]	Animal study	Effects of PBMT on cardiac fibrosis activation post-MI	Experimental MI induction, PBMT application (660 nm, 15 mW, 22.5 J/cm2, 60 s, 0.785 cm2, 1.1 J/cm2) post-coronary artery ligation; ventricular septal samples collected at 30 min, 3, 6, 24 hours, 3 days post-MI to assess mRNA and miRNA expression	
Feliciano et al. 2021 [36]	Animal study	PBMT's effect on transcriptional & post-transcriptional changes post-MI	660 nm CW non-thermal laser (15 mW, 0.9 J, 1.15 J/cm2, 0.785 cm2, 60 s) for PBMT; in silico analysis to select 47 genes from 9 MI-related signaling pathways; mRNA expression quantification in myocardial samples by PCR real-time array using TaqMan customized plates; global miRNA expression analysis	
Gao et al. 2022 [37]	Animal study	PBM's effect on cardiac physiological activity	Noninvasive irradiation of mice with 630 nm LED-Red light; investigation of cardiomyocyte (CM) division, proliferation, and intracellular photopower; myocardial revascularization, regeneration, and fibrosis reduction in MI mice; miRNA sequencing analysis for CMs; luciferase reporter assays for miR-136-5p and Ino80 binding; Ino80 expression and knockdown experiments	
Grandinetti et al. 2019 [38]	Animal study	Combined PBMT and carvedilol treatment in infarcted rats	Infarcted rats treated with carvedilol and PBMT for 30 days; functional fitness evaluated using a motorized treadmill; echocardiography and hemodynamic measurements for left ventricular (LV) functional evaluations; ELISA, Western blot, and biochemical assays to evaluate inflammation and oxidative stress in the myocardium	
Hentschke et al. 2013 [39]	Animal study	LLLT's effect on inflammation in rats with heart failure	Induction of heart failure in male Wistar rats (n=49) by ligating the left coronary artery; rats were assigned to six groups: placebo sham, LLLT at 3 J/cm(2) sham, LLLT at 21 J/cm(2) sham, placebo HF, LLLT at 3 J/cm(2) HF, and LLLT at 21 J/cm(2) HF; LLLT (InGaAlP 660 nm, spot size 0.035 cm(2), output power 20 mW, power density 0.571 W/cm(2), energy density 3 or 21 J/cm(2), exposure time 5.25 s and 36.75 s) applied to right gastrocnemius for 10 consecutive days, 4 weeks after myocardial infarction or sham surgery	
Lohr et al. 2009 [24]	Animal study	Red/Near Infrared light's role in NO release and cardioprotective effects	Examined if R/NIR light could facilitate the release of NO from nitrosyl heme proteins, and if R/NIR light could enhance the protective effects of nitrite on ischemia and reperfusion injury in rabbit hearts; studied the effects of R/NIR light on purified systems and myocardium	
Malinovskaya et al. 2008 [40]	Animal study	Effects of light irradiation on rat hearts after ischemia	Conducted experiments on 91 male albino rats; induced ischemia by occluding the left coronary artery for 5 minutes; randomized rats into 2 control and 2 experimental groups; experimental group 1 received laser irradiation, experimental group 2 received wideband red light; irradiation started immediately after removal of the ligature and lasted for 10 min; measured ECG, LPO products, and SOD activity	
Manchini et al. 2017 [25]	Animal study	Effects of LLLT on post-infarction cardiac remodeling	Female Wistar rats subjected to coronary occlusion to induce myocardial infarction or Sham operation; single LLLT application carried out 60 s and 3 days post-coronary occlusion; echocardiography performed 3 days and at 5 weeks to evaluate cardiac function; LV hemodynamic evaluation performed at baseline and on sudden afterload increases; myocardial expression of AKT1/VEGF pathway analyzed	
Manchini et al. 2014 [41]	Animal study	Effects of LLLT on inflammation and cardiac function post-MI	Female rats subjected to acute myocardial infarction (MI); LLLT treatment applied; MI size, systolic dysfunction, myocardial mRNA expression of interleukin-1 beta and interleukin-6, protein and mRNA levels of the Mas receptor, mRNA expression of kinin B1 and B2 receptors, plasma kallikrein, vascular endothelial growth factor (VEGF) expression, capillaries density, inducible nitric oxide synthase (iNOS) and endothelial NOS mRNA content, and plasma nitric oxide metabolites (NOx) concentration evaluated	
Syed et al. 2023 [28]	Animal study	Effects of PBM therapy on age-associated cardiovascular changes	14-month-old AC8 overexpressing transgenic mice (n=8) and WT littermates (n=8) treated with daily Near-Infrared Light (850 nm) at 25 mW/cm2 for 2 min each weekday for a total dose of 1 Einstein (4.5 p.J/cm2 or fluence 3 J/cm2) for 8 months; PBM therapy was administered for 3.5 months (Early Treatment period), paused for 3 months due to Covid-19 restrictions, and restarted for 1.5 months; serial echocardiography and gait analyses were performed at monthly intervals, and serum TGF-β1 levels were assessed following sacrifice	
Tuby et al. 2006 [42]	Animal study	Effect of LLLT on VEGF and iNOS expression in infarcted hearts	Myocardial infarction induced by occlusion of the left descending artery in 87 rats; LLLT was applied to intact and post-infarction hearts; VEGF, iNOS, and angiogenesis were determined	
Wang et al. 2019 [43]	Animal study	Effect of LED therapy on AMI-induced ventricular Arrhythmias (VA), microglia, and sympathetic activation	30 anesthetized rats randomly divided into 3 groups: Control (n=6), AMI (n=12), and AMI+LED (n=12); ECG and left stellate ganglion (LSG) neural activity were continuously recorded; Incidence of VAs recorded during the first hour after AMI; Brain and myocardium tissue samples examined for microglial activation and expression of NGF, IL-18, and IL-1β	
Yang et al. 2011 [26]	Animal study	Effect of LLLI on cardiac cytokine expression and ventricular remodeling after MI	MI created by coronary ligation; Surviving rats divided into laser (n=33) and control (n=33) groups; Laser group exposed to a diode laser; Control group received coronary ligation only; 28 rats received thoracotomy without coronary ligation (sham group); Cytokine antibody arrays, ELISA, echocardiography, and histological studies performed	

In this comprehensive review, the therapeutic potential of energy-based modalities, specifically low-level laser therapy (LLLT) and photobiomodulation (PBM), for cardiovascular disorders is investigated. A total of 19 studies were examined, and their findings are summarized as follows.

I. Effects of LLLT/PBM on cardiac function and remodeling

Several studies have explored the effects of Low-Level Laser Therapy (LLLT) and Photobiomodulation (PBM) on cardiac function and remodeling following myocardial infarction (MI). Biasibetti et al. [29] revealed that LLLT altered oxidative balance in skeletal muscle of heart failure (HF) rats by reducing superoxide dismutase (SOD) activity and dichlorofluorescein (DCFH) oxidation levels. However, high LLLT doses led to increased DNA damage [29]. Meanwhile, Blatt et al. [30] established that applying LLLT to bone marrow resulted in diminished scarring, enhanced angiogenesis, and functional improvement following MI in a porcine model [30]. Bublitz et al. [31] concluded that while LLLT did not enhance functional capacity in HF patients, it potentially modulated blood lactate metabolism and decreased perceived muscle fatigue [31].

Capalonga et al. [32] demonstrated that light-emitting diode therapy (LEDT) elevated functional capacity in heart failure (HF) rats, as evidenced by improved distance, time, and speed during exercise [32]. Feliciano et al. [35] discovered that photobiomodulation therapy (PBMT) reversed alterations in myocardial extracellular matrix gene mRNA expression, modified cardiac microRNAs (miRNAs) expression associated with fibrosis replacement, and identified correlations between specific miRNAs and mRNA [35]. Feliciano et al. [36] in their 2021 study found that MI led to modified mRNA expression of various biomarkers linked to detrimental cardiac activity, and PBMT reverted most of these transcriptional changes, particularly decreasing mRNA expression of IL-6, tumor necrosis factor (TNF) receptor, transforming growth factor β 1 (TGF-β1), and collagen I and III. PBMT also reduced miR-221, miR-34c, and miR-93 expression post-MI [36].

Gao et al. [37] determined that LED-Red promoted cardiomyocyte (CM) division and proliferation, myocardial revascularization, and regeneration while reducing fibrosis area in MI mice, improving cardiac contractile function. MiR-136-5p was identified as a cardiac photo-sensitive miRNA with proliferation-promoting effects, and INO80 as a miR-136-5p binding target in CM proliferation regulation [37]. Grandinetti et al. [38] showed that carvedilol and PBMT comparably ameliorated pulmonary congestion, left ventricle (LV) end-diastolic pressure, LV dilation, and LV systolic function. PBMT combined with carvedilol inhibited myocardial hypertrophy, improved +dP/dt of LV, and exhibited superior anti-inflammatory effects [38]. Manchini et al. [41] reported that LLLT diminished MI size, attenuated systolic dysfunction [41].

Syed et al. [28] discovered that early PBM treatments reduced age-associated increases in left ventricular (LV) mass, decreased LV end-diastolic volume (EDV) in AC8, lowered left atrial dimension, enhanced LV ejection fraction, alleviated aortic wall stiffness, and improved gait symmetry. These effects persisted after the pause, and cumulative survival increased in PBM-treated AC8 mice. PBM treatment was found to mitigate age-associated cardiovascular remodeling, reduce cardiac function, enhance neuromuscular coordination, and increase longevity in an experimental animal model, with responses correlating with elevated TGF-β1 in circulation [28].

However, some studies reported contradictory results. For instance, Manchini et al. [25] in their 2017 study found that the beneficial effects of LLLT on left ventricular (LV) systolic function may be dependent on the maintenance of phototherapy [25]. LLLT reduced MI size, attenuated systolic dysfunction, and decreased myocardial mRNA expression of interleukin-1 beta and interleukin-6, as reported by Manchini et al. [41] in 2014, but did not show significant changes in vascular endothelial growth factor (VEGF) expression or capillaries' density [41]. More research is needed to confirm these findings and to determine the optimal dose and wavelength of LLLT for disease treatment.

II. Anti-inflammatory effects of LLLT/PBM

The anti-inflammatory effects of LLLT/PBM have been demonstrated in various studies, showcasing their potential in mitigating inflammation in injured cardiovascular tissues. Hentschke et al. [39] found that LLLT reduced plasma IL-6 levels, TNF-α/IL-10, and IL-6/IL-10 ratios while increasing IL-10 levels in rats with heart failure [39]. Manchini et al. [25] in his 2017 study observed that laser light treatment influenced numerous biomarkers associated with inflammation and myocardial repair, although no significant changes were detected in VEGF expression or capillary density [25]. Wang et al. [43] revealed that LED therapy significantly attenuated microglial activation and reduced IL-18, IL-1β, and nerve growth factor (NGF) expression in the peri-infarct myocardium [43].

III. Effects of LLLT/PBM on angiogenesis

Blatt et al. [30] established that applying LLLT to bone marrow resulted in enhanced angiogenesis [30]. Tuby et al. [42] demonstrated that laser-irradiated rat hearts post-infarction and intact hearts exhibited a significant increase in VEGF and inducible nitric oxide synthase (iNOS) expression compared to non-laser-irradiated hearts. LLLT also caused a significant elevation in angiogenesis, and the upregulated VEGF and iNOS expression in the infarcted rat heart was associated with enhanced angiogenesis and cardioprotection [42].

IV. Other effects of LLLT/PBM

Derkacz et al. [33] observed that LLLT contributed to reduced TGF-β1 and fibroblast growth factor-2 (FGF-2) levels, consequently leading to smaller neointima formation, decreased late lumen loss, and a lower restenosis rate [33]. In a subsequent study, Derkacz et al. [34] reported not only elevated nitrite/nitrate concentrations but also a transient increase in endothelin-1 in the laser group, which was accompanied by a reduced restenosis rate [34]. Lohr et al. [24] elucidated that R/NIR light could decay nitrosyl hemes and release NO, thereby augmenting the cardioprotective effects of nitrite [24]. Furthermore, Malinovskaya et al. [40] highlighted that wideband red light irradiation resulted in decreased mortality compared to laser irradiation and control groups, restored heart rate, diminished lipid peroxidation (LPO) products, and increased SOD activity in myocardial tissues [40]. Additionally, Wang et al. [43] unveiled that LED therapy significantly reduced the incidence of acute myocardial infarction (AMI)-induced ventricular arrhythmias (VAs) and decreased left stellate ganglion (LSG) neural activity in the AMI+LED group compared to the AMI group. Of note, LED therapy significantly attenuated inflammatory cytokine expression in the peri-infarct myocardium, suggesting a potential protective effect against AMI-induced VAs through the suppression of sympathetic neural activity and the inflammatory response [43]. Lastly, Yang et al. [26] employed a cytokine antibody array to identify cytokines involved in the response to therapeutic laser irradiation, finding that low-level laser irradiation (LLLI) did not improve damaged heart function but reduced infarct area expansion [26].

Despite showing promising results in pre-clinical investigations, it is important to note that the small sample sizes and varied methodological approaches of available literature may be the Achilles' heel of this study, making it difficult to draw definitive conclusions. The studies were also of limited scope because they had a short follow-up period and were conducted in different animal models and human populations. The studies also used different doses and wavelengths of LLLT. As a result, the long-term safety and efficacy of LLLT could not be assessed. The studies were conducted in different animal models, including mice, rats, and rabbits. The studies used different doses of LLLT, ranging from 1 to 22 J/cm2. The studies also used different wavelengths of LLLT, ranging from 630 to 900 nm. The results may vary depending on the type of animal model, the human population, the dose of LLLT, and the wavelength of LLLT. More research is needed to determine the long-term safety and efficacy of LLLT.

Future implications

The studies discussed here demonstrate the potential of photobiomodulation therapy (PBMT) in treating various aspects of heart failure and acute myocardial infarction. Although the mechanisms of action of PBMT are still not fully understood, it is clear that it has a beneficial effect on several biomarkers and processes related to cardiac function and remodeling. The findings suggest that PBMT could be used in combination with traditional pharmacological therapies to enhance their effects or as a standalone treatment for patients who are not responsive to or cannot tolerate traditional therapies.

Moreover, future studies should focus on optimizing PBMT protocols, including the timing, frequency, and duration of treatments, as well as the use of different types of light sources and wavelengths. Furthermore, PBM has been used to delay the presentation of age-related cardiac disorders. A recent study by Syed et al. [28] suggests that PBM therapy could mitigate age-associated cardiovascular remodeling and improve cardiac function, neuromuscular coordination, and longevity in an experimental animal model. Additionally, the observed responses correlated with increased TGF-β1 levels in circulation [28]. These findings indicate that PBM therapy may have promising benefits in preventing or slowing cardiovascular aging and may serve as a potential therapeutic strategy for age-related cardiovascular diseases in the future.

Finally, larger-scale randomized controlled trials are needed to validate the findings of these studies and to investigate the long-term safety and efficacy of PBMT in different patient populations.

## Conclusions

In conclusion, the studies discussed in this article suggest that PBMT has promising potential in the treatment of heart failure and acute myocardial infarction. The mechanisms of action of PBMT appear to involve anti-inflammatory, anti-fibrotic, and pro-angiogenic effects, which could help mitigate the downward spiral of heart failure and promote tissue repair and regeneration. While further research is needed to fully understand the mechanisms of action of PBMT, and its potential adverse effects and to optimize treatment protocols, the findings to date are encouraging and suggest that PBMT could be a valuable addition to the armamentarium of therapies available for heart failure and acute myocardial infarction.

## Appendices

Supplementary data

PubMed Search Strategy

Search strategy: ("Low-Level Light Therapy"[Mesh]) AND ("Cardiovascular Diseases"[Mesh] OR "Cardiology"[Mesh])

382 results

Google Scholar

94 results

Central

130 results
